# Supplementary figures and images for: Comprehensive characterization of the Hsp70 interactome reveals novel client proteins and interactions mediated by posttranslational modifications
Source: PLoS Biol. 2022 Oct 21;20(10):e3001839. doi: 10.1371/journal.pbio.3001839 (PMC9629621; doi:10.1371/journal.pbio.3001839)

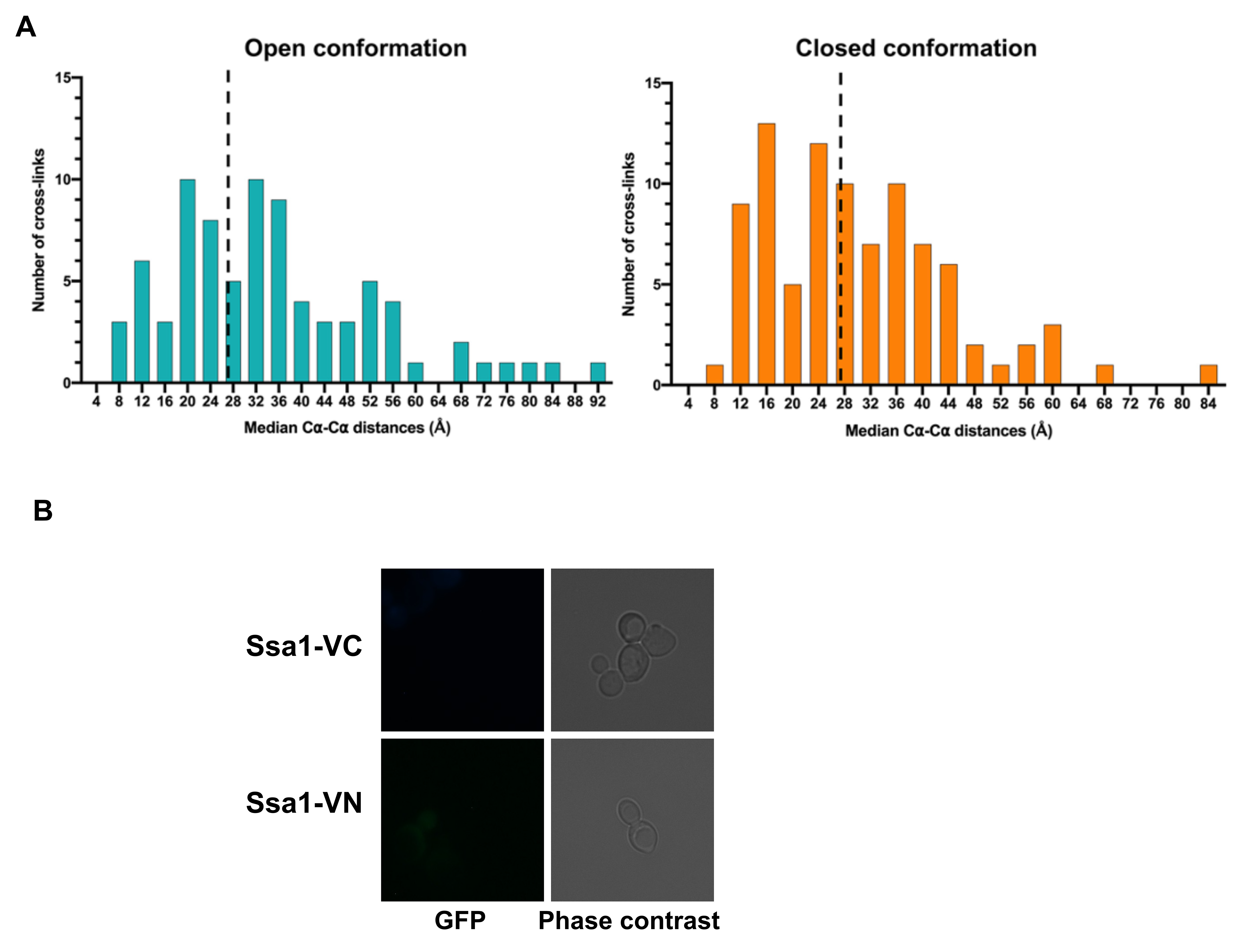

Supplement: S1 Fig — (A) Distances between cross-linked amino acids on identified Ssa-Ssa1 peptides based on monomeric Hsp70 in open and close conformations. Maximum allowed cross-link length of DSSO is represented as a dashed line. All cross-linked peptides where the calculated distance between 2 Ssa1 residues is greater than the DSSO spacer arm cannot come from a single Ssa1 molecule. (B) Control for Ssa1-Ssa1 BiFC experiment. Yeast expressing either only VN-Ssa1 or VC-Ssa1 were analyzed for BiFC signal. The data underlying the graphs shown in the figure can be found in S1 Data. (TIF) [file pbio.3001839.s001.tif]

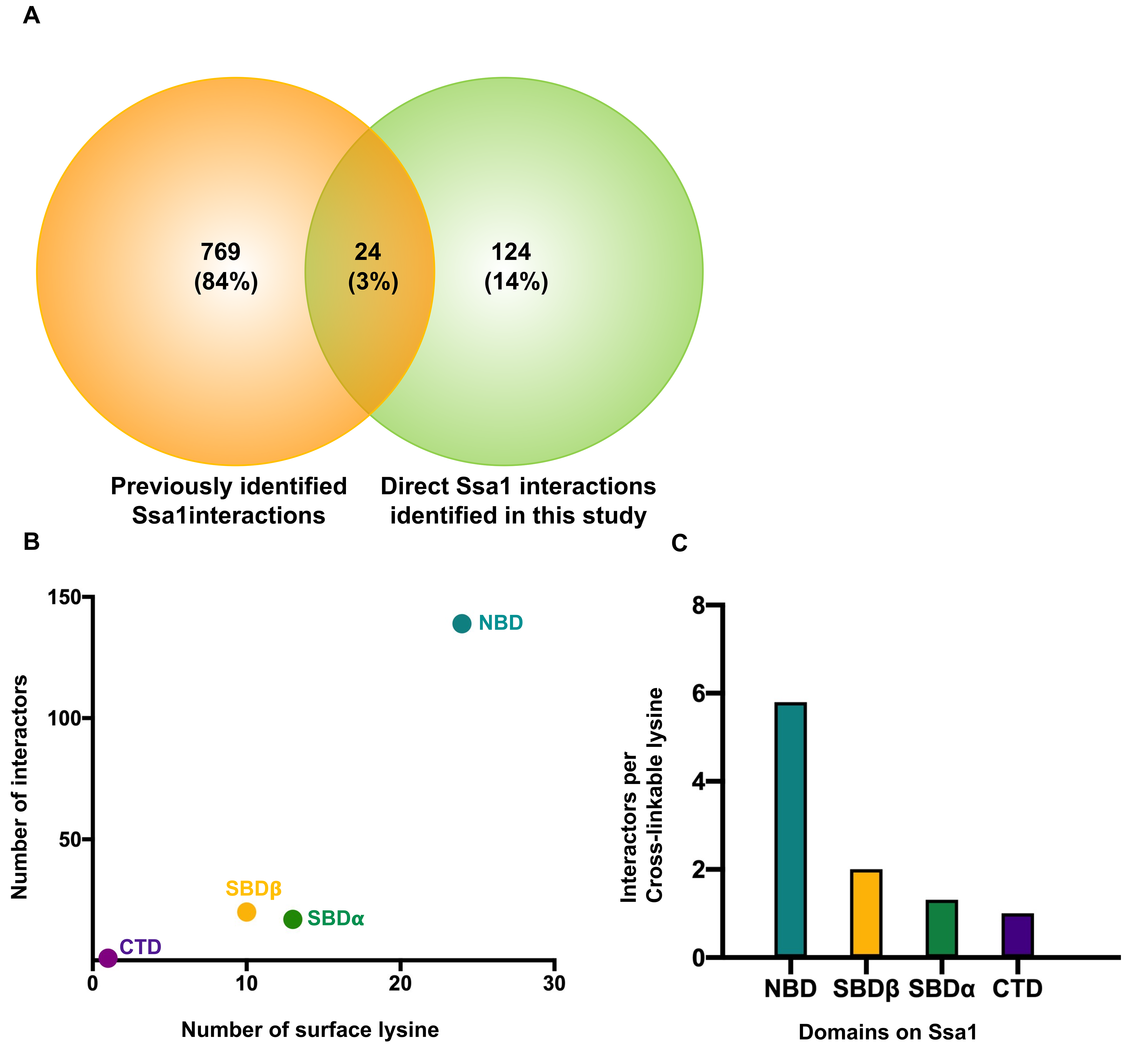

Supplement: S2 Fig — (A) Venn diagram representing previously known physical interactors of Ssa1 versus direct interactors of Ssa1 identified in this study. (B) Scatter plot of number of interactors identified versus surface lysine on the domains of Ssa1. (C) Bar graph representing interactors per cross-linkable lysine on domains of Ssa1. The data underlying the graphs shown in the figure can be found in S1 Data. (TIF) [file pbio.3001839.s002.tif]

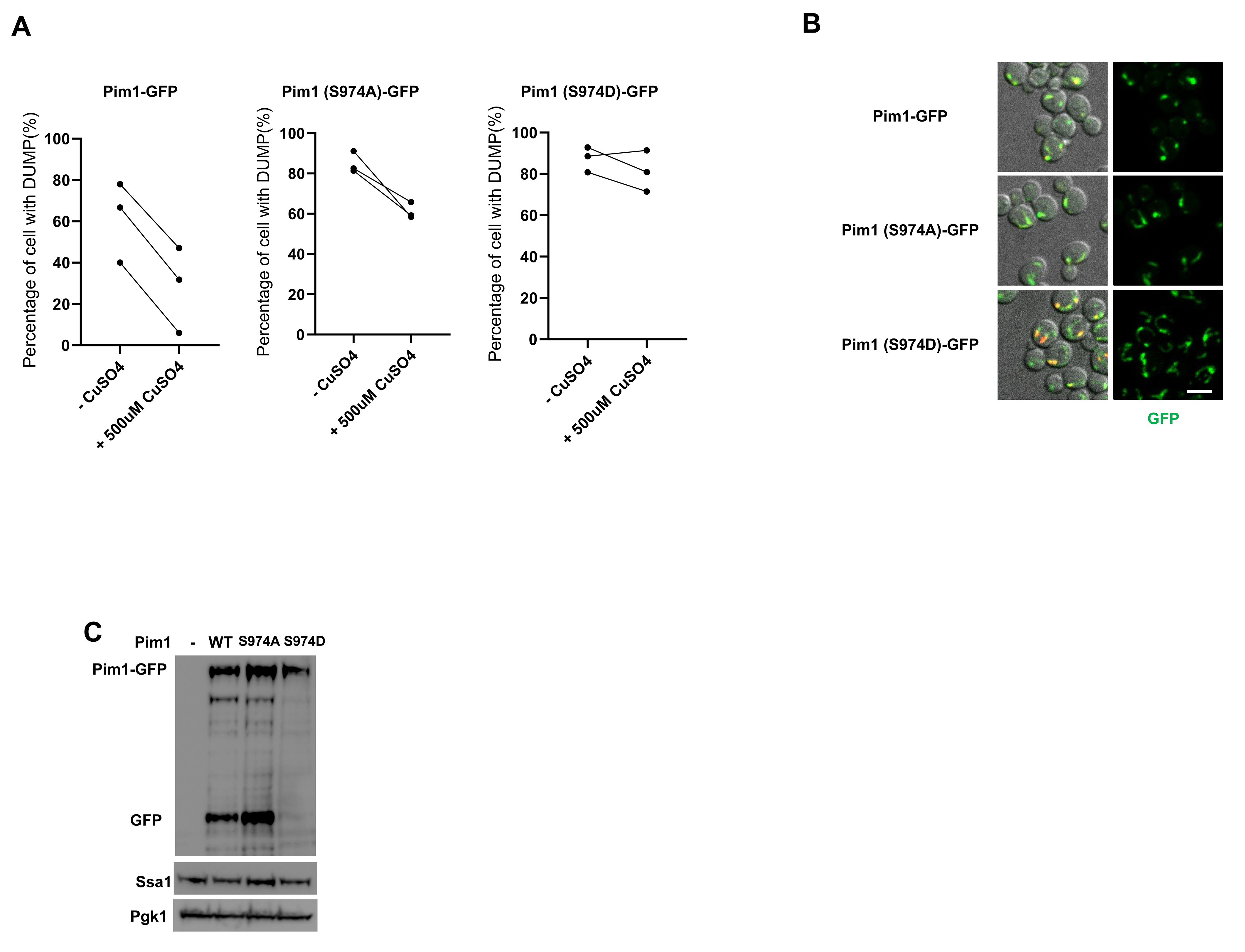

Supplement: S3 Fig — (A) Quantification of percentage of cell with mitoFluc labeled DUMP structures in (Fig 5G). (B). Localization of Pim1 wild type and the mutants in yeast cells. Paired t test was used for statistical analysis. (C) Western blot showing the levels of Pim1 wild type and the mutants in yeast cells. The data underlying the graphs shown in the figure can be found in S1 Data. (TIF) [file pbio.3001839.s003.tif]

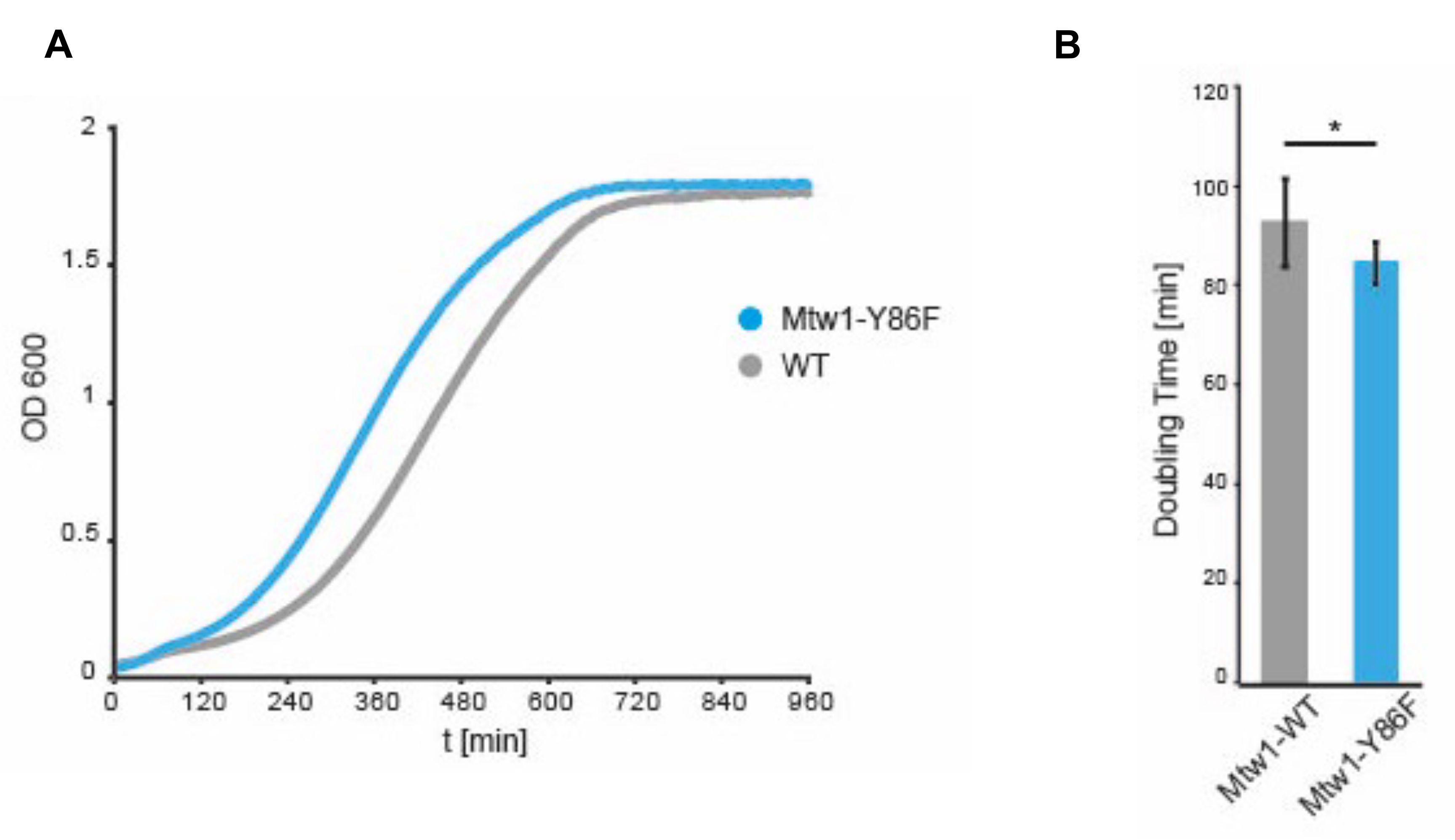

Supplement: S4 Fig — (A) Growth curves of wild-type (BY4741) and Mtw1-Y86F yeast strains. Cells were diluted from an overnight culture to OD600 = 0.03 and the OD600 was measured every 5 min for 16 h using a microplate reader. Growth analysis was performed in 10 replicates per strain. (B) Doubling times for wild-type and mutant strains were calculated for each replicate and compared using Student t test (mean = 92.97 min, p-value = 0.04). The data underlying the graphs shown in the figure can be found in S1 Data. (TIF) [file pbio.3001839.s004.tif]
