# Supplementary material for: Comprehensive characterization of the Hsp70 interactome reveals novel client proteins and interactions mediated by posttranslational modifications
Source: PLoS Biol. 2022 Oct 21;20(10):e3001839. doi: 10.1371/journal.pbio.3001839 (PMC9629621; doi:10.1371/journal.pbio.3001839)
Supplement: S1 Raw Images — (PPTX) [file pbio.3001839.s007.pptx]

## Slide 1
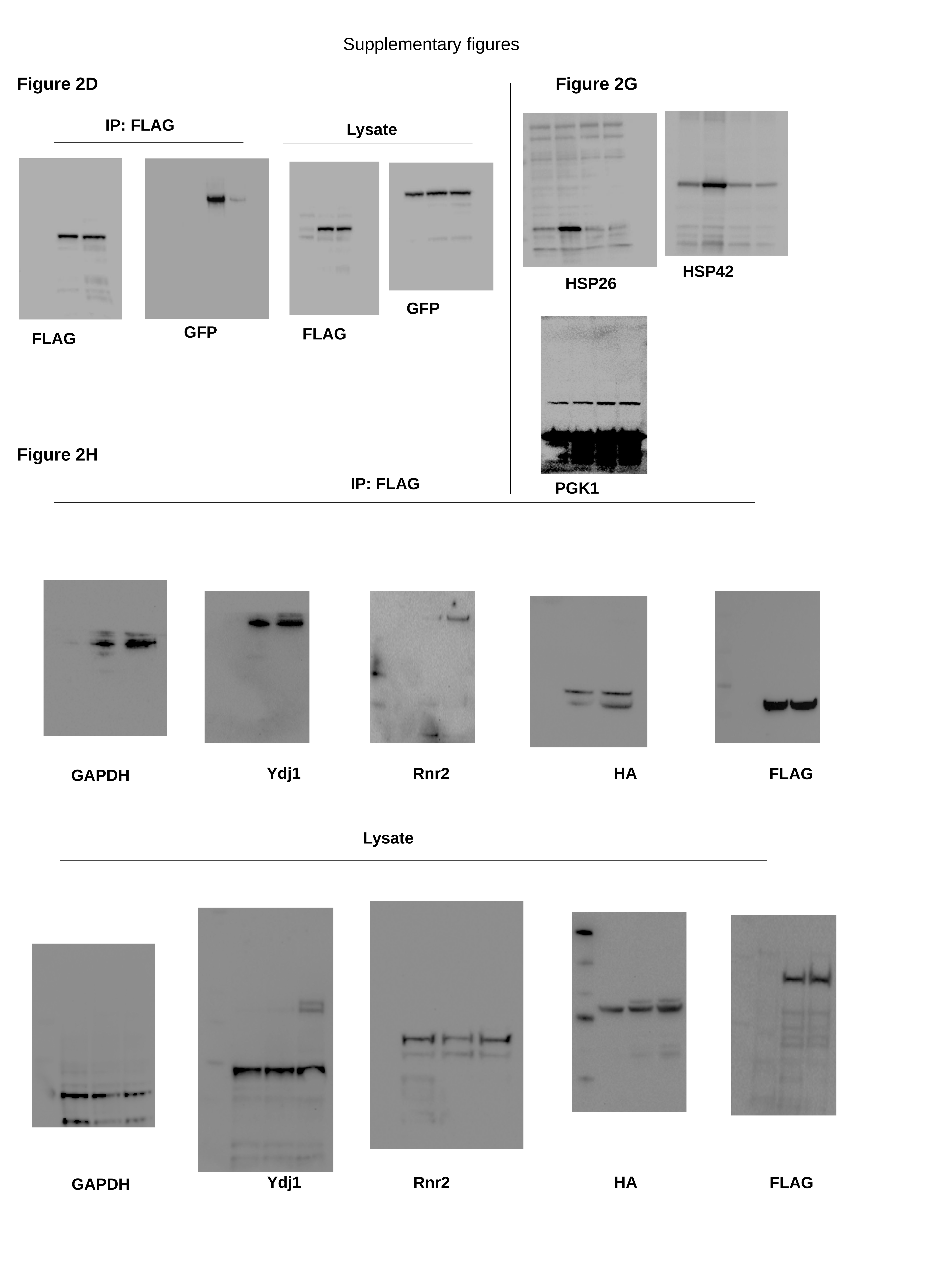

Supplementary figures
Figure 2D
Figure 2G
IP: FLAG
Lysate
 HSP42
 HSP26
 GFP
 GFP
 FLAG
 FLAG
Figure 2H
IP: FLAG
PGK1
Ydj1
HA
Rnr2
FLAG
GAPDH
Lysate
Ydj1
HA
Rnr2
FLAG
GAPDH

## Slide 2
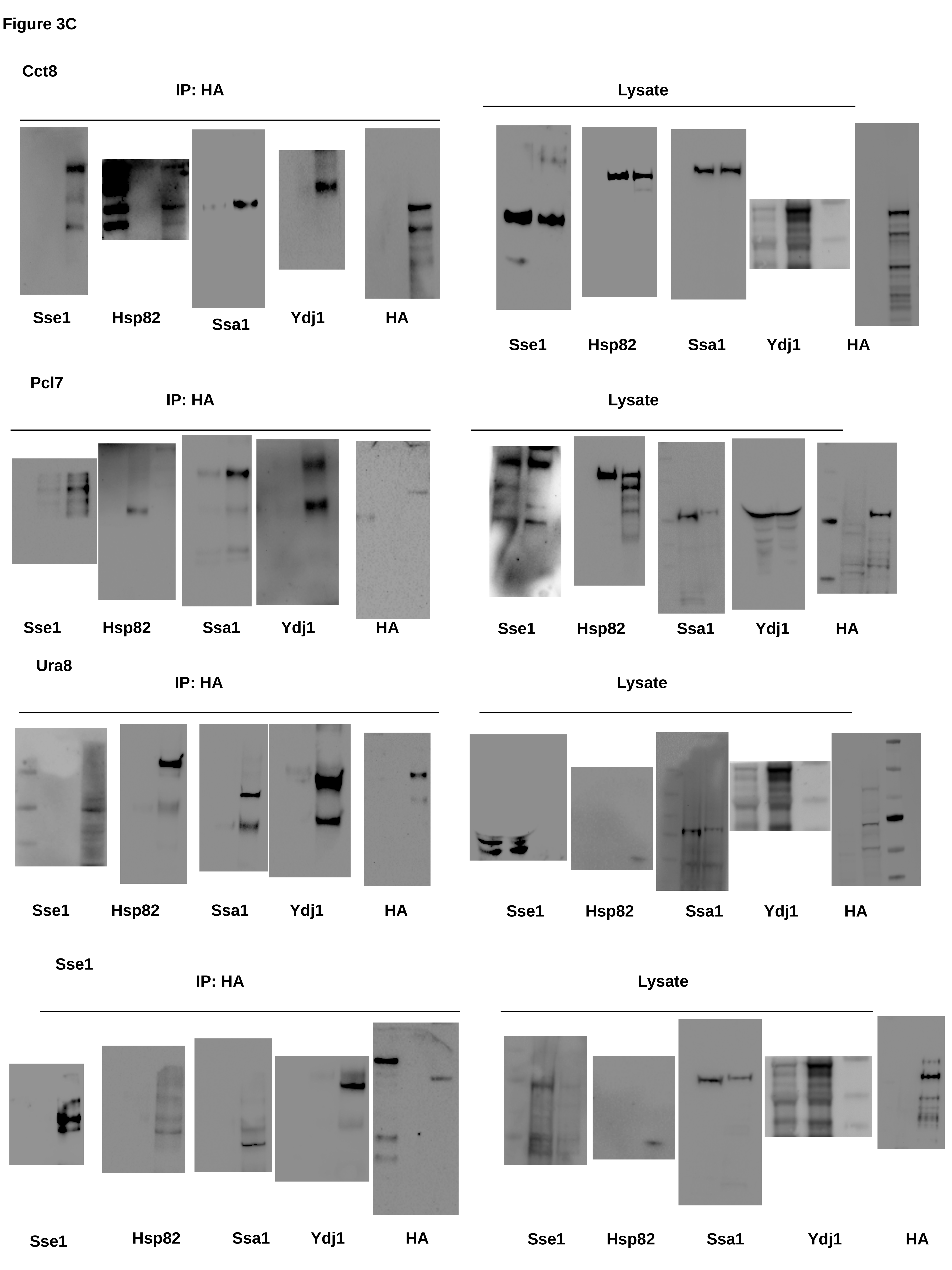

Figure 3C
Cct8
IP: HA
Lysate
Sse1
Hsp82
Ydj1
HA
Ssa1
Sse1
Hsp82
Ssa1
Ydj1
HA
Pcl7
IP: HA
Lysate
Sse1
Hsp82
Ssa1
Ydj1
HA
Sse1
Hsp82
Ssa1
Ydj1
HA
Ura8
IP: HA
Lysate
Sse1
Hsp82
Ssa1
Ydj1
HA
Sse1
Hsp82
Ssa1
Ydj1
HA
Sse1
IP: HA
Lysate
Hsp82
Ssa1
Ydj1
HA
Sse1
Hsp82
Ssa1
Ydj1
HA
Sse1

## Slide 3
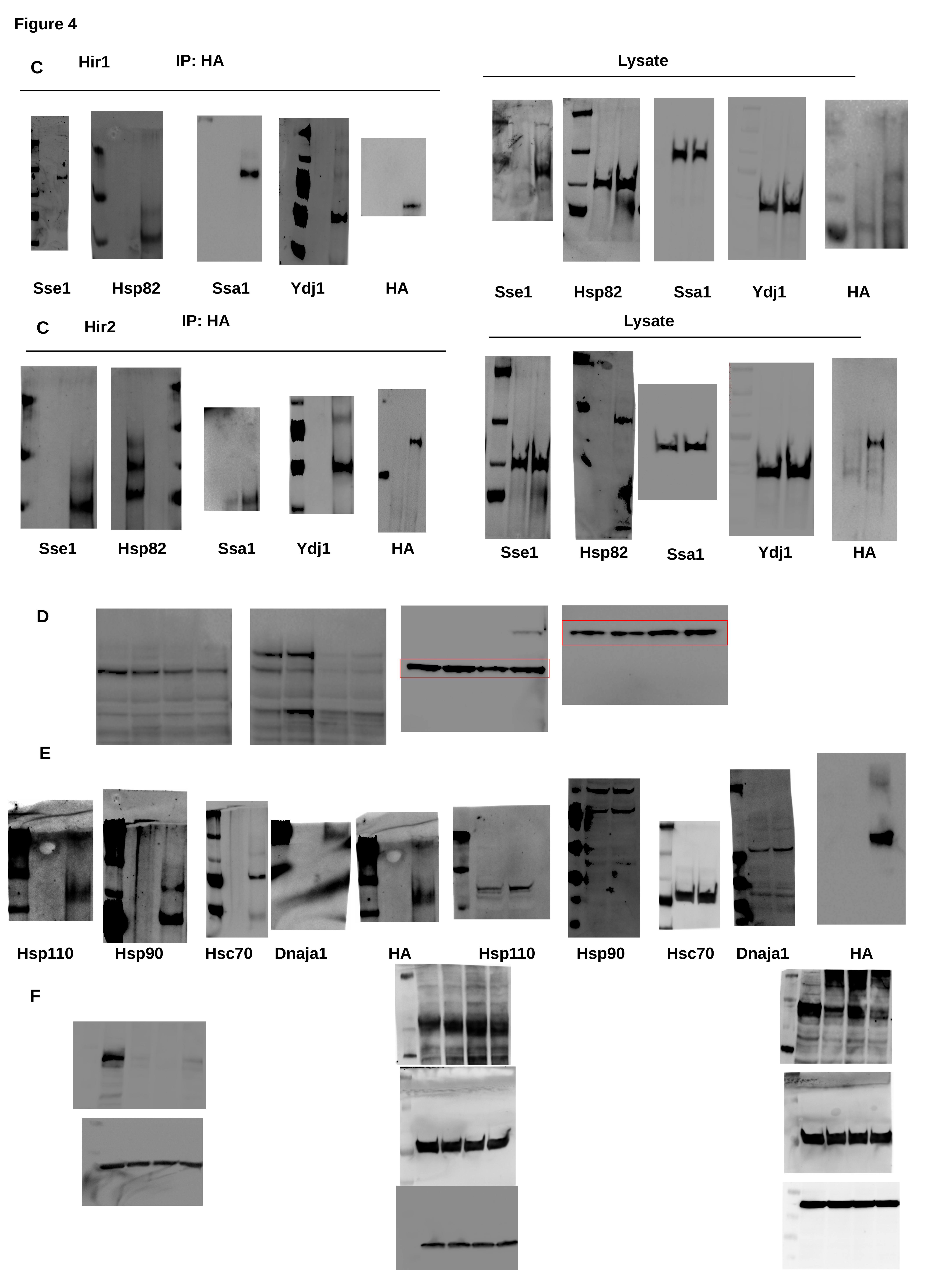

Figure 4
IP: HA
Lysate
Hir1
C
Sse1
Hsp82
Ssa1
Ydj1
HA
Sse1
Hsp82
Ssa1
Ydj1
HA
IP: HA
Lysate
C
Hir2
Sse1
Hsp82
Ssa1
Ydj1
HA
Sse1
Hsp82
Ydj1
HA
Ssa1
D
E
Hsp110
Hsp90
Hsc70
Dnaja1
HA
Hsp110
Hsp90
Hsc70
Dnaja1
HA
F

## Slide 4
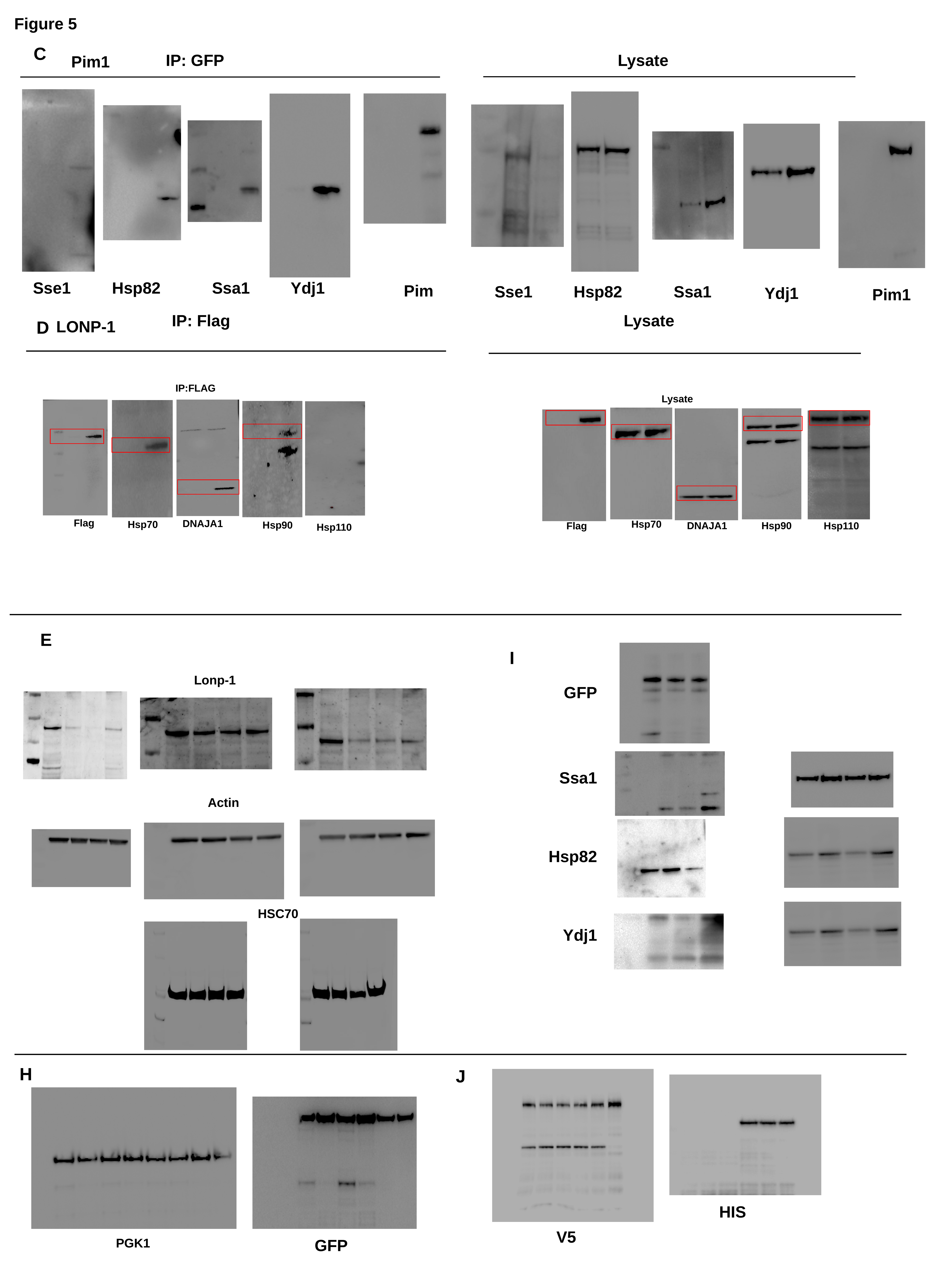

Figure 5
C
IP: GFP
Lysate
Pim1
Sse1
Hsp82
Ssa1
Ydj1
Pim
Sse1
Hsp82
Ssa1
Ydj1
Pim1
IP: Flag
Lysate
D
LONP-1
IP:FLAG
Flag
DNAJA1
Hsp70
Hsp90
Hsp110
Lysate
Hsp70
DNAJA1
Hsp90
Flag
Hsp110
E
I
Lonp-1
GFP
Ssa1
Actin
Hsp82
HSC70
Ydj1
H
J
HIS
V5
PGK1
GFP

## Slide 5
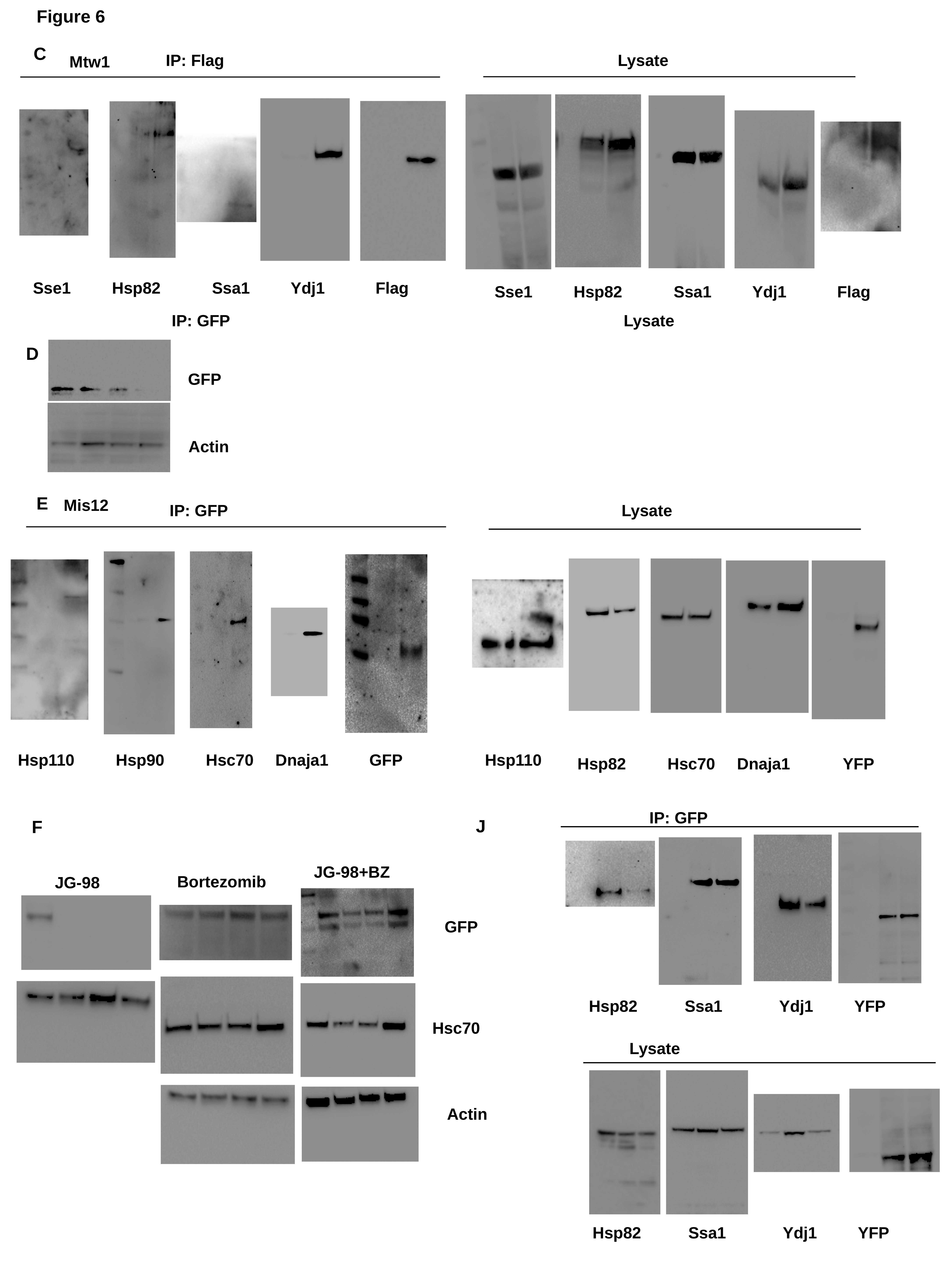

Figure 6
C
IP: Flag
Lysate
Mtw1
Sse1
Hsp82
Ssa1
Ydj1
Flag
Sse1
Hsp82
Ssa1
Ydj1
Flag
IP: GFP
Lysate
D
GFP
Actin
E
Mis12
IP: GFP
Lysate
Hsp110
Hsp90
Hsc70
Dnaja1
GFP
Hsp110
Hsp82
Hsc70
Dnaja1
YFP
IP: GFP
J
F
JG-98+BZ
Bortezomib
JG-98
GFP
Hsp82
Ssa1
Ydj1
YFP
Hsc70
Lysate
Actin
Hsp82
Ssa1
Ydj1
YFP

## Slide 6
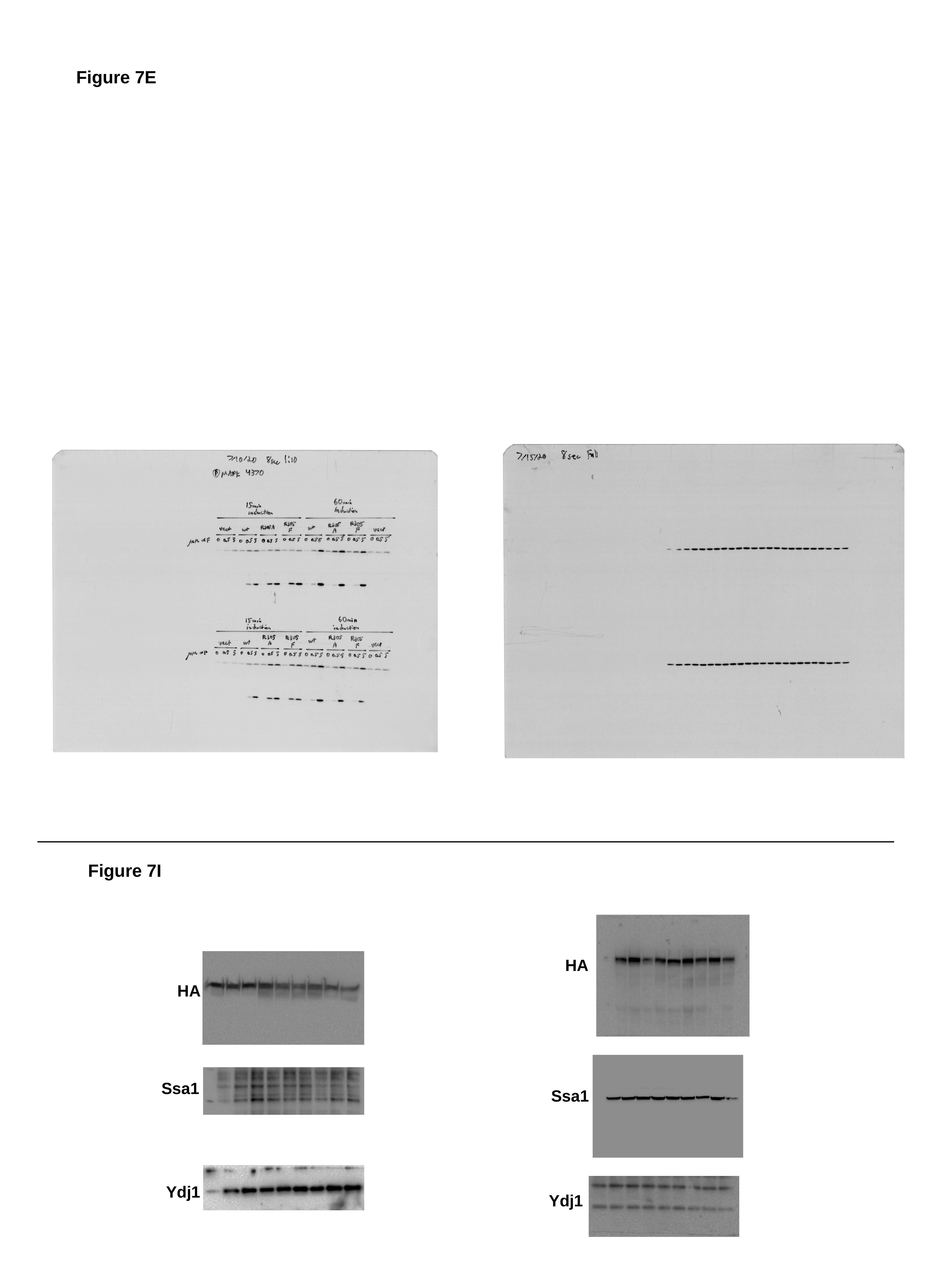

Figure 7E
Figure 7I
HA
HA
Ssa1
Ssa1
Ydj1
Ydj1

## Slide 7
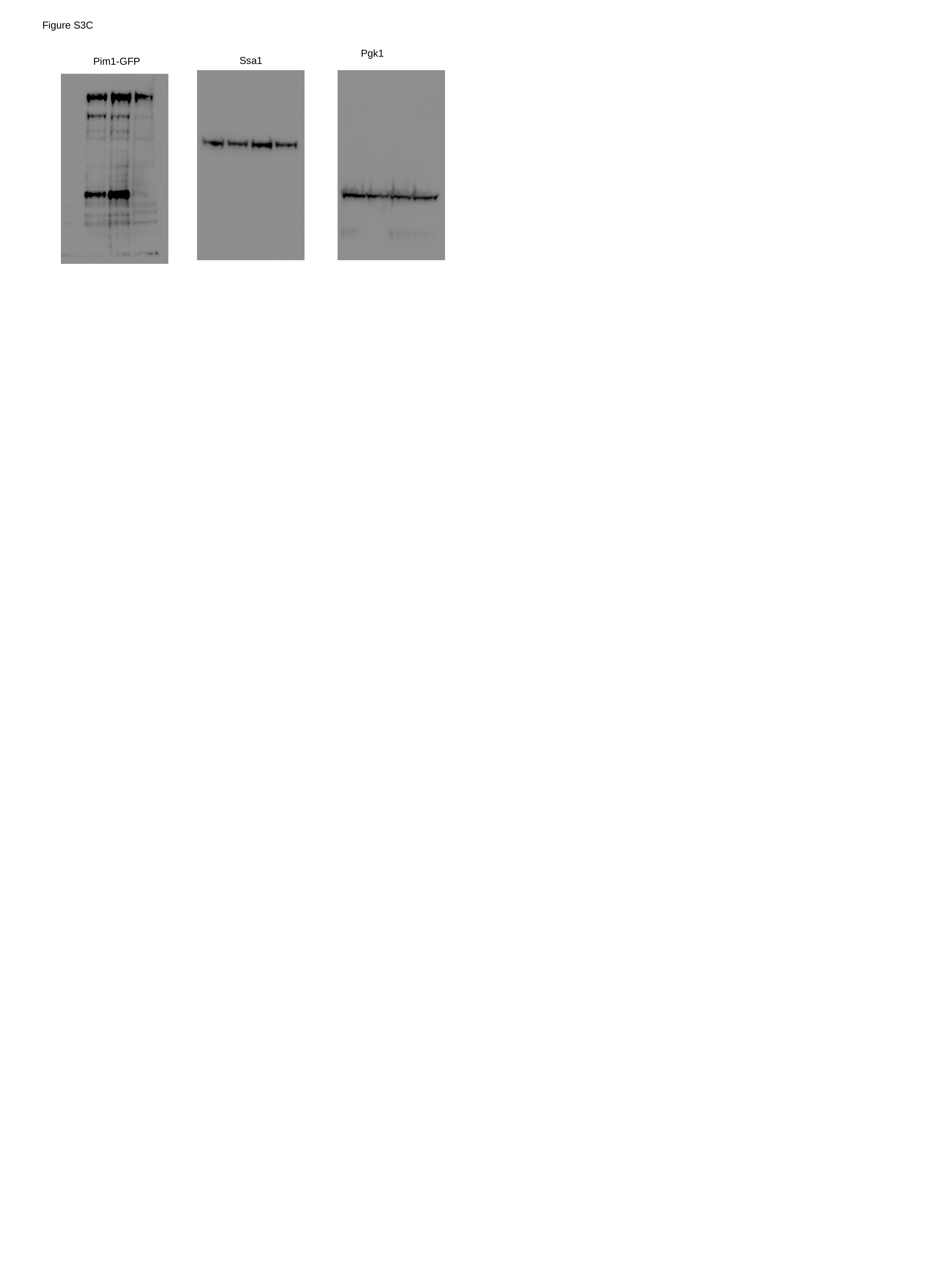

Figure S3C
Pgk1
Ssa1
Pim1-GFP
