# Supplementary material for: Comprehensive characterization of the Hsp70 interactome reveals novel client proteins and interactions mediated by posttranslational modifications
Source: PLoS Biol. 2022 Oct 21;20(10):e3001839. doi: 10.1371/journal.pbio.3001839 (PMC9629621; doi:10.1371/journal.pbio.3001839)
Supplement: Attachment — Submitted filename: Reviewer response for PLOS Biology R1 Aug 12.pdf [file pbio.3001839.s009.pdf]

UNIVERSITY OF NORTH CAROLINA CHARLOTTE | COLLEGE OF LIBERAL ARTS & SCIENCES BIOLOGICAL SCIENCES

Department of Biological Sciences
9201 University City Boulevard, Woodward 486C
Charlotte, NC 28223, USA

Tel: (443) 248-3287
e-mail: atruman1@uncc.edu
Website: www.trumanlab.org
Twitter: @trumanlab

June 16, 2022

Dear Editor,

We offer below our point-by-point responses to queries and comments on our submitted manuscript previously titled, “A novel multifunctional role for Hsp70 in binding post-translational modifications on client proteins” (now retitled “Cross-linking mass spectrometry analysis of Hsp70 complexes reveal novel PTM-associated interactions”). We would like to thank yourself and the reviewers for guidance and constructive comments. This work was initially started just before the pandemic hit the senior author graduated after the submission of this paper. Despite this, we have done our best to answer the reviewer queries. We have been able to complete most of the requested experiments and have adjusted the text to better reflect the data presented.

Before we go into the point-by-point responses, we wanted to address here some general queries. We would ask the reviewers to keep in mind that this work is being considered as a ***Resource*** article. As per the *PLOS Biology* website, “Resources consist of data sets or other significant scientific resources that are of general interest and provide exceptional value for the community that could spur future research”. We would ask the reviewers and Editor to compare our revised manuscript with [1], a smaller resources paper published in *PLOS Biology* in 2020 on a related, yet distinct theme and methodology. We set out to identify novel <u>direct</u> interactors of Hsp70 by using cross-linking mass spectrometry, something that has not been attempted previously. We recruited proteomic experts Dr. Luca Fornelli (University of Oklahoma) and Dr. Romain Huguet (Thermo, Mass Spectrometry section). We designed and analyzed the proteomics experiments with appropriate controls and statistics to ensure an extremely high degree of rigor. Through this work, we have identified novel chaperone interactions, many of which appear to be associated with PTMs (95% of these PTMs were previously unknown). Aside from the interest to the general chaperone community, the newly discovered PTMs on a range of proteins will be interesting to the scientific community as a whole.

In accordance with reviewer suggestions, we have been careful to remove the term “client” throughout the manuscript where client status has not been fully investigated. We have also altered the title to “Cross-linking mass spectrometry analysis of Hsp70 complexes reveal novel PTM-associated interactions” to reflect this change.

Our work (now including requested data) suggests that Ssa1 self-association is important for a subset of its chaperone functions, particularly the heat shock response. We revised this section in particular for clarity and insight. While much more work needs to be done to explore all of these fascinating interactions, we believe these data will be of great benefit to the

chaperone community (and beyond) and thus fulfills the criteria for a *Resources* article in *PLOS Biology*.

Response to Reviewer 1 comments

"*A major absence is the experiment showing that the E540A/N537K mutants do not dimerize - this is inferred but not tested despite the reagents being in hand.*"

We agree with the reviewer. We have completed the recommended experiment and the new data can be found as Fig. 2D.

"*Fig. 1G - I believe the N537A nomenclature is incorrect as the results text reads N537K*"

Yes, it should be N537A and we have made the appropriate changes to Fig. 1G and the text.

"*Fig. 1G and 1H - the temperature sensitive phenotype is indeed quite dramatic and an attempt is made to link the growth phenotype to a reduced HSR. While this is possible, it is not consistent with past results that suggest that impaired HSR does not typically result in ts growth but rather heat shock sensitivity, which could be easily tested*.

Defects in yeast Hsf1 activity are known to result in yeast temperature sensitivity. A good example if this is the well-characterized *hsf1-583* mutant [2-4].

"*More importantly, it would be useful to assess Hsf1 target protein levels in the strains to ascertain if chaperone levels are truly significantly reduced in the mutant strains. I suspect they are not*."

We thank the reviewer for this suggestion. We examined the inducibility of two Hsf1-driven proteins (Hsp26 and Hsp42) in WT and N537A/E540A strain via Western Blotting. There was a very clear defect in Hsp26 and Hsp42 inducibility in the mutant. This taken together with the *ts* phenotype and HSE-luciferase assays clearly shows that there is an issue with activation of the HSF in N537A/E540A. The new figure can be found as Figure 2G. We have revised the appropriate section in the results and discussion sections of the manuscript in accordance with this.

"*Finally, the corresponding author is well aware that chaperone and Hsf1 mutants are typically ts due to failure to maintain the cell wall stress response. This should also have been tested using sorbitol plates as done later in the manuscript to ask whether osmotic support suppresses the ts phenotype.*"

This is a fair point by the reviewer-the corresponding author has published several papers linking the HSR to cell wall integrity. We have completed the proposed sorbitol experiment which can now be found in a revised Fig. 1E. There is a clear suppression of the N537A/E540A *ts* phenotype, adding to evidence that N537A/E540A has a defective heat shock response, similar that of the *hsf1-583* mutant [2-4].

"*Fig. 1I - why is the load control protein GAPDH present in the FLAG-Ssa1 IP?*

This result may appear strange, but GAPDH is actually a known interactor, but not a client of Ssa1 [1]. We decided to use this as a control for our experiment using Ssa1 V435F. As expected, V435F Ssa1 still interacts with GAPDH confirming its non-client status.

"*As an extension of comment #4, the paper relies heavily on differential signals by immunoblot for protein levels and pulldowns, yet no quantitation of these blots is provided".*

While quantitation is useful in some studies, we do not think this is the case here. In the experiments demonstrating novel protein interaction with Ssa1, it is a binary result (either interacting or not). For drug inhibition assays, there is a clear decrease in client levels upon chaperone inhibition. We believe in this case the addition of graphs showing blot quantitation would not only make the figures less readable but would be an unnecessary time and cost burden for the researchers involved.

"*Fig. 3 C - what is the purpose of the cartoon of a yeast cell above the pulldowns? What is happening in the lysates for Hsp82?"*

The purpose of the cartoon yeast was to clarify which experiments were performed in yeast vs cell culture. We agree this was a bit confusing and inconsistent and have removed this from the figures."

With regards to Hsp82 lysate levels, we agree that it is interesting that Hsp82 levels are altered in response to overexpression of Sse1 and Ura8. We saw this on multiple attempts of this experiment. Hsp82 is a stress-induced protein and it is possible that overexpression of these client proteins is triggering the production of Hsp82. For the purposes of this experiment, we simply sought to demonstrate Hsp82-interaction with these potential clients, which we believe I clear from our presented data.

"Fig. 5D - what is being pulled down here - the results say Lonp-1 is the bait while the figure suggests it is FLAG-Hsc70."

The reviewers are quite correct, the original experiment used FLAG-Hsc70 as the bait. On reflection, we realized a much better experiment would be to use FLAG-Lonp-1 as the bait. We have completed this experiment which can now be found in revised figure 5D.

"It is unclear what is actually happening in Fig. 5H and 5J. Does bortezomib inhibit the lon protease? If so, a citation would be useful to support that fact."

We apologize for the lack of clarity on this. Yes, several studies have shown Bortezomib does inhibit Lon protease processing. In addition to this, the crystal structure of yeast, bacterial and mammalian lon protease with bortezomib has been characterized [5-9]. We have now added these references to the manuscript.

"In 5J, the results state that self-cleavage was restored in the S974D mutant. However, it appears that cleavage is actually blocked in the asparate mutant in the presence of Ssa1 - am I misinterpreting the banding patterns here?".

With regards to the S974 mutant the reviewer is correct-cleavage is actually blocked in the asparate mutant in the presence of Ssa1 when expressed in bacteria. We apologize for this error and have corrected this in the text.

"Is Ssa1 associated with cytosolic Pim1 prior to mitocondrial insertion? No comments are made regarding the organellar nature of this client."

This is an interesting point from the reviewer. Pim1 functions in the mitochondria whereas Ssa1 is typically found in the cytoplasm. Our collaborator Dr. Rong Li, (a world leader in Pim1/Lonp-1 function) believes that based on our data the Ssa1-Pim1 interaction is occurring in the cytoplasm, preventing Pim1 from inappropriately digesting cytoplasmic targets. Release of Pim1 by Ssa1 into the mitochondria would allow it to perform its appropriate function. Experimentally, this is very complex to decipher, but we have altered the manuscript discussion to discuss this potential mechanism in more detail.

"Fig. 7 - much of the results in this figure appear to show no real effects of the mutations and should either be moved to the SI or the authors should consider removing the figure entirely - there's not a lot going on here and it ends the paper on a less-than-compelling note."

Ste11 was one of the first identified clients of the Hsp90 system in yeast and as such we were excited to see this protein as a direct interactor of Ssa1. The regulation of the Ste11 pathway is complex involving multiple layers of regulation. The modification on Ste11 (di-methylation on R305) has not been previously identified. To try and tease out the role of this site we recruited two leading experts in Ste11 pathway regulation, Drs. Tatebayashi and Pryciak, who were very excited by our XL-MS data (given the proximity of R305 to activatory phosphorylation sites). Although subtle, their data do clearly show an impact of R305 mutation on Ste11 function. This modification is particularly challenging to tease apart as there are no good antibodies for di-methylation modification. This may be addressed in future studies using in vitro dimethylation or expanded genetic code technologies, but we have done the best we could given the available reagents. Given that this is a *resources* article, we believe identification of this site as an interesting regulatory site for Ste11 is enough for publication.

"Why is the presence of Hsp82 investigated in most if not all of the interactions? The positive results here suggest that these are actually clients of the greater Hsp90 system, which of course include Hsp70 as a precursor step. It is also unusual for both Hsp90 and Hsp70/Ydj1 to be present at the same time, so the pulldowns likely represent mixed populations of clients with several different chaperones."

We agree with the reviewer that the nature of the MS experiment means that we are likely pulling down mixed populations of clients and that many of these interactions are likely to be clients of the greater Hsp90 system. However, although Hsp70 and Hsp90 have distinct roles in the folding process, they are often seen together in complexes. Examples include studies from the Wickner lab which demonstrate that Hsp70 and Hsp90 can directly interact [10, 11] and multiple high-profile studies from the Agard lab on the glucocorticoid receptor which forms a complex of both Hsp90 and Hsp70 at the same time [12, 13].

Response to Reviewer 2

"Nitika et al provide an in vivo interactive analysis of the Hsp70 chaperone Ssa1, using cross-linking mass spectrometry. If correct, this study is absolutely awesome"

We thank the review for the kind comments, it means a lot to the students and faculty involved.

"Almost 80% of the interactions are located in the nucleotide binding domain (NBD). While it is plausible that regulatory factors interact with the NBD, it not plausible that such interactions outnumber substrate interactions by 4:1. In fact, I would have even expected more interactions in the substrate binding domain than identified by the authors. This raises the questions

whether the interactions monitored here are relevant and really reflect meaningful interactions inside the cell. Ssa1 is a rather abundant protein, equipped with a cross linker it may interact with many proteins. Would overproduction of NBD lead to a similar interactive? Would it me toxic, by competing out the interactions? Would V435F be toxic? How does the interactive of this mutant compare to wildtype?"

The whole purpose of this work was to identify *direct* interactors of Hsp70 as opposed to those in a complex. We have over 10 years of experience analyzing chaperone interactions by mass spectrometry and have published these in well-respected journals [14-20]. For this project, we were fortunate enough to also recruit collaborators Dr. Luca Fornelli (an expert in carrying out and the analysis of XL-MS experiments) and Dr. Romain Huguet (who works for Thermo pioneering new MS technologies). From a technical perspective, appropriate steps were taken to ensure a high level of confidence in the MS data. All the interactions we have followed up on so far have proven to be genuine interactors through complementary technologies.

We agree with the reviewers that it is unexpected that many of the interactions seen are with the N-terminus as opposed to the C-terminus, given that the C-terminus is defined as the client-binding region. There are several potential explanations for these results. Firstly, it is possible that during the client protein-binding process, there are multiple interactions between chaperone and client-protein that engage the entirety of Hsp70. Secondly, it is likely that we are detecting the interaction of Hsp70 in fully-formed protein complexes, such as observed in [12, 13]. Finally, several of the N-terminal interactions may represent novel co-chaperones/regulators of Hsp70, which we hope to investigate in future studies. From a technical perspective, the imbalance of interactions observed between the N and C-terminal domains of Ssa1 may arise from the cross-linker used. Hsp70 has a client binding preference consisting of short stretches of amino acids enriched in hydrophobic and non-polar amino acids, with a model client peptide of NRLLLTG often being used in *in vitro* studies [48]. DSSO is a lysine cross-linker and it is possible that the lack of lysines on clients at the Ssa1 client-binding domain interface may prevent DSSO linkage [26]. It is also worth noting that the substrate-binding domain on Ssa1 is heavily modified by PTMs, many of which occur on surface lysines [21]. The presence of these PTMs on the SBD may inhibit DSSO-cross linking in this region.

Experimentally, this is hard to tease out. A lack of interaction between the isolated Hsp70 NBD and a protein does not disprove that an interactor can bind the NBD. This result could also be explained by a model in which the protein can interact with both NBD and SBD, but this is dependent on the SBD. We considered performing single domain experiments in yeast but the NBD by itself is not enough to sustain yeast viability. If the NBD were expressed in yeast in the presence of full-length Ssa1, results would be complicated by the ability of Ssa1 to either oligomerize or NBD-SBD interactions that happen as part of the Ssa1 conformational cycle. We have however made substantial edits to the manuscript to discuss these points, including a removal of the term "client" from most of the figures and text. We hope that this satisfies the reviewers.

"The authors find many interactions for the dimerisation of Hsp70. When analysing substrate interactions, they only observe single cross-links. This suggests that concentration indeed plays a major role for the findings of the study. E.g. Hir1 shows only 1 cross link to the SBD, Pim1 only 1 cross link to the NBD."

We agree with the reviewer, but it should be noted observation of single crosslinks are pretty standard in the XL-MS field even in highly purified recombinant protein complexes (Please see [21-23]).

"*Pim1 is a mitochondrial protease. Why should it now interact with the NBD of Hsp70*?"

After identifying interaction between Ssa1 and Pim1, we recruited Dr. Rong Li, an expert in Pim1/LonP from Johns Hopkins University to study this interesting interaction. With her help, the data we have collected suggests that the Ssa1 binds Pim1, preventing it from self-cleaving, becoming active and inappropriately digesting cytoplasmic targets. Release of Pim1 by Ssa1 into the mitochondria would allow it to perform its native function. While our data suggests that Pim1 is a direct interactor and client of Ssa1, the fact that we observe a cross-link only at the N-terminus of Ssa1 does not of course mean the interaction is exclusively through this region. It is likely that Ssa1 and Pim1 interact at multiple points, but that the nature of the experiment and kinetics of interaction did not allow us to capture these other interactions. We have altered the manuscript discussion to discuss this potential mechanism in more detail and why many more interactions with NBD than SBD were observed.

"*For the analysis of dimerisation, the question is to which extent does dimerisation play a role - how much of Hsp70 is present in the dimer? if the interaction is meaningful under stress, what would be its physiological role? Dimerisation had been suspected in the literature as a storage function (although never proven in vivo), but this would expect that under heat stress Hsp70 should more monomers to interact with unfolded clients*."

There is substantial evidence from mammalian and bacterial systems that Hsp70 homodimerizes/oligomerizes (please see our review [24]). Qinglian Liu's lab obtained the structure of a DnaK dimer and impaired DnaK function via mutagenesis of dimer-disrupting sites [25]. Human Hsp70 has also been observed as anti-parallel dimers (at least *in vitro*) by cross-linking mass spectrometry, native electrospray mass spectrometry and small-angle X-ray scattering based studies [12, 26]. In addition, ER-localized Hsp70 (BiP) has been known to form dimers under stress conditions [27-29].

We now show with additional experiments suggested by the reviewers that mutation of amino acids in Ssa1 that are conserved and are critical for DnaK and human Hsc70 dimerization appear to prevent Ssa1 self-association. These same mutations prevent yeast from mounting an appropriate heat shock response. This includes temperature sensitivity that can be suppressed by sorbitol, a loss of HSE-luciferase activity and induction of Hsp26/42. While beyond the scope of this *Resources* article we hypothesize that Ssa1 dimerization alters interaction with the core heat shock machinery rather than unfolded protein. We hope to investigate this further in future studies. We apologize for not explaining this well and have substantially edited the text for clarity.

Response to Reviewer 3

"*The manuscript was written in a readable and accessible style, and the data are beautifully and clearly presented. I have some minor suggestions for the authors to consider during revision of the manuscript.*"

We thank the reviewer for their kind comments. This means a lot to all the researchers involved.

"*Figure 1A describes the core of the methodology but is hardly mentioned in the narrative at all. It is left implicit that the reader will figure out the approach from the figure. It would be useful to the reader to provide a concise but informative narrative to describe the broad approach in the text.*"

We apologize for this error and have added detail to the methods section as requested.

Fig 2F: there are no controls for the BiFc analysis. As a minimum, the untagged VN and VC should be shown for the reader to have confidence in the data.

We agree and have added this data as supplemental figure S1B.

"*Fig 2G-H: Would it be possible to conclusively demonstrate loss of dimerisation using the IP or BiFc approach as reported by the authors for example*?"

We agree with the reviewer's comment. We have now performed an experiment demonstrating that the mutant does not dimerize to the same extent as WT Ssa1. This figure can be found as revised Fig. 2D

"*The authors use the term 'client' quite loosely when describing the interactome analysis. My understanding, which is reinforced by the authors themselves later in the ms, is that interaction alone is not sufficient to infer client status. A client is one that interacts and is reliant on the chaperone (i.e. is perturbed upon chaperone inhibition). Therefore, it would be more accurate to use the term "interactor" or similar unless where the authors demonstrate client status".*

We absolutely agree with the point. While we have demonstrated novel client-like behavior for a few proteins, the others are untested. We have removed "client" in the text where true client behavior has not been demonstrated. To that end we also changed the title of the manuscript accordingly.

"*Fig 4 D: the - and + signs are not correctly aligned with the lanes"*

Fixed

"Figure 5H and I: should it be Pim1-GFP?"

Fixed

"Fig 6H and I: How certain are you at the increased localization to the kinetochore of the YFP tagged protein is not just due to higher levels of protein expression? There did appear to be high levels of diffuse fluorescence in the background?"

The Y86 site lies in the interface between Mtw1 and other core kinetochore components [30, 31] and thus likely impacts Mtw1 interactions. Given monetary and time constraints we were unable to experimentally address this, but instead have edited the manuscript to discuss this in more detail.

"Page 14, line 5: 'previous' should be 'previously'"

Fixed

"The methods section needs to be reviewed for formatting and consistency. There are several minor issues that should be easily resolved."

We apologize for these mistakes and now have corrected the figures and text as requested.

We hope that these responses and the revised manuscript are satisfactory. Again, we thank the Editor and Reviewers for their careful reading and valuable feedback. We feel this process has led to a vastly improved manuscript.

Sincerely,

Andrew Truman, PhD
Associate Professor, Biological Sciences, University of North Carolina at Charlotte

References

1. Ryu SW, Stewart R, Pectol DC, Ender NA, Wimalarathne O, Lee JH, et al. Proteome-wide identification of HSP70/HSC70 chaperone clients in human cells. PLoS Biol. 2020;18(7):e3000606. Epub 20200720. doi: 10.1371/journal.pbio.3000606. PubMed PMID: 32687490; PubMed Central PMCID: PMCPMC7392334.
2. Morano KA, Santoro N, Koch KA, Thiele DJ. A trans-activation domain in yeast heat shock transcription factor is essential for cell cycle progression during stress. Mol Cell Biol. 1999;19(1):402-11. doi: 10.1128/MCB.19.1.402. PubMed PMID: 9858564; PubMed Central PMCID: PMCPMC83898.
3. Truman AW, Millson SH, Nuttall JM, Mollapour M, Prodromou C, Piper PW. In the yeast heat shock response, Hsf1-directed induction of Hsp90 facilitates the activation of the Slt2 (Mpk1) mitogen-activated protein kinase required for cell integrity. Eukaryot Cell. 2007;6(4):744-52. Epub 20070209. doi: 10.1128/EC.00009-07. PubMed PMID: 17293484; PubMed Central PMCID: PMCPMC1865661.
4. Sorger PK. Yeast heat shock factor contains separable transient and sustained response transcriptional activators. Cell. 1990;62(4):793-805. doi: 10.1016/0092-8674(90)90123-v. PubMed PMID: 2201452.
5. Bayot A, Basse N, Lee I, Gareil M, Pirotte B, Bulteau AL, et al. Towards the control of intracellular protein turnover: mitochondrial Lon protease inhibitors versus proteasome inhibitors. Biochimie. 2008;90(2):260-9. Epub 20071025. doi: 10.1016/j.biochi.2007.10.010. PubMed PMID: 18021745.
6. Csizmadia V, Hales P, Tsu C, Ma J, Chen J, Shah P, et al. Proteasome inhibitors bortezomib and carfilzomib used for the treatment of multiple myeloma do not inhibit the serine protease HtrA2/Omi. Toxicol Res (Camb). 2016;5(6):1619-28. Epub 20160825. doi: 10.1039/c6tx00220j. PubMed PMID: 30090462; PubMed Central PMCID: PMCPMC6062231.
7. Yang J, Song AS, Wiseman RL, Lander GC. Cryo-EM structure of hexameric yeast Lon protease (PIM1) highlights the importance of conserved structural elements. J Biol Chem. 2022;298(3):101694. Epub 20220207. doi: 10.1016/j.jbc.2022.101694. PubMed PMID: 35143841; PubMed Central PMCID: PMCPMC8913295.
8. Shin M, Puchades C, Asmita A, Puri N, Adjei E, Wiseman RL, et al. Structural basis for distinct operational modes and protease activation in AAA+ protease Lon. Sci Adv. 2020;6(21):eaba8404. Epub 20200520. doi: 10.1126/sciadv.aba8404. PubMed PMID: 32490208; PubMed Central PMCID: PMCPMC7239648.
9. Shin M, Watson ER, Song AS, Mindrebo JT, Novick SJ, Griffin PR, et al. Structures of the human LONP1 protease reveal regulatory steps involved in protease activation. Nat Commun. 2021;12(1):3239. Epub 20210528. doi: 10.1038/s41467-021-23495-0. PubMed PMID: 34050165; PubMed Central PMCID: PMCPMC8163871.
10. Kravats AN, Hoskins JR, Reidy M, Johnson JL, Doyle SM, Genest O, et al. Functional and physical interaction between yeast Hsp90 and Hsp70. Proc Natl Acad Sci U S A. 2018;115(10):E2210-E9. Epub 20180220. doi: 10.1073/pnas.1719969115. PubMed PMID: 29463764; PubMed Central PMCID: PMCPMC5877982.
11. Genest O, Wickner S, Doyle SM. Hsp90 and Hsp70 chaperones: Collaborators in protein remodeling. J Biol Chem. 2019;294(6):2109-20. Epub 20181106. doi: 10.1074/jbc.REV118.002806. PubMed PMID: 30401745; PubMed Central PMCID: PMCPMC6369297.
12. Morgner N, Schmidt C, Beilsten-Edmands V, Ebong IO, Patel NA, Clerico EM, et al. Hsp70 forms antiparallel dimers stabilized by post-translational modifications to position clients for transfer to Hsp90. Cell Rep. 2015;11(5):759-69. Epub 20150423. doi: 10.1016/j.celrep.2015.03.063. PubMed PMID: 25921532; PubMed Central PMCID: PMCPMC4431665.

13. Wang RY, Noddings CM, Kirschke E, Myasnikov AG, Johnson JL, Agard DA. Structure of Hsp90-Hsp70-Hop-GR reveals the Hsp90 client-loading mechanism. Nature. 2022;601(7893):460-4. Epub 20211222. doi: 10.1038/s41586-021-04252-1. PubMed PMID: 34937942; PubMed Central PMCID: PMCPMC9179170.
14. Xu L, Nitika, Hasin N, Cuskelly DD, Wolfgeher D, Doyle S, et al. Rapid deacetylation of yeast Hsp70 mediates the cellular response to heat stress. Sci Rep. 2019;9(1):16260. Epub 20191107. doi: 10.1038/s41598-019-52545-3. PubMed PMID: 31700027; PubMed Central PMCID: PMCPMC6838335.
15. Knighton LE, Nitika, Waller SJ, Strom O, Wolfgeher D, Reitzel AM, et al. Dynamic remodeling of the interactomes of Nematostella vectensis Hsp70 isoforms under heat shock. J Proteomics. 2019;206:103416. Epub 20190621. doi: 10.1016/j.jprot.2019.103416. PubMed PMID: 31233900; PubMed Central PMCID: PMCPMC7304457.
16. Woodford MR, Truman AW, Dunn DM, Jensen SM, Cotran R, Bullard R, et al. Mps1 Mediated Phosphorylation of Hsp90 Confers Renal Cell Carcinoma Sensitivity and Selectivity to Hsp90 Inhibitors. Cell Rep. 2016;14(4):872-84. Epub 20160121. doi: 10.1016/j.celrep.2015.12.084. PubMed PMID: 26804907; PubMed Central PMCID: PMCPMC4887101.
17. Truman AW, Kristjansdottir K, Wolfgeher D, Ricco N, Mayampurath A, Volchenboum SL, et al. Quantitative proteomics of the yeast Hsp70/Hsp90 interactomes during DNA damage reveal chaperone-dependent regulation of ribonucleotide reductase. J Proteomics. 2015;112:285-300. Epub 20141018. doi: 10.1016/j.jprot.2014.09.028. PubMed PMID: 25452130; PubMed Central PMCID: PMCPMC4485990.
18. Dunn DM, Woodford MR, Truman AW, Jensen SM, Schulman J, Caza T, et al. c-Abl Mediated Tyrosine Phosphorylation of Aha1 Activates Its Co-chaperone Function in Cancer Cells. Cell Rep. 2015;12(6):1006-18. Epub 20150730. doi: 10.1016/j.celrep.2015.07.004. PubMed PMID: 26235616; PubMed Central PMCID: PMCPMC4778718.
19. Truman AW, Kristjansdottir K, Wolfgeher D, Hasin N, Polier S, Zhang H, et al. CDK-dependent Hsp70 Phosphorylation controls G1 cyclin abundance and cell-cycle progression. Cell. 2012;151(6):1308-18. Epub 2012/12/12. doi: 10.1016/j.cell.2012.10.051. PubMed PMID: 23217712; PubMed Central PMCID: PMCPMC3778871.
20. Mollapour M, Tsutsumi S, Truman AW, Xu W, Vaughan CK, Beebe K, et al. Threonine 22 phosphorylation attenuates Hsp90 interaction with cochaperones and affects its chaperone activity. Mol Cell. 2011;41(6):672-81. Epub 2011/03/23. doi: 10.1016/j.molcel.2011.02.011. PubMed PMID: 21419342; PubMed Central PMCID: PMCPMC3062913.
21. Liu F, Lossl P, Rabbitts BM, Balaban RS, Heck AJR. The interactome of intact mitochondria by cross-linking mass spectrometry provides evidence for coexisting respiratory supercomplexes. Molecular & cellular proteomics : MCP. 2018;17(2):216-32. Epub 20171208. doi: 10.1074/mcp.RA117.000470. PubMed PMID: 29222160; PubMed Central PMCID: PMCPMC5795388.
22. Klykov O, Steigenberger B, Pektas S, Fasci D, Heck AJR, Scheltema RA. Efficient and robust proteome-wide approaches for cross-linking mass spectrometry. Nat Protoc. 2018;13(12):2964-90. Epub 2018/11/18. doi: 10.1038/s41596-018-0074-x. PubMed PMID: 30446747.
23. Liu F, Rijkers DT, Post H, Heck AJ. Proteome-wide profiling of protein assemblies by cross-linking mass spectrometry. Nature methods. 2015;12(12):1179-84. Epub 20150928. doi: 10.1038/nmeth.3603. PubMed PMID: 26414014.
24. Takakuwa JE, Nitika, Knighton LE, Truman AW. Oligomerization of Hsp70: Current Perspectives on Regulation and Function. Front Mol Biosci. 2019;6(81):81. Epub 20190904. doi: 10.3389/fmolb.2019.00081. PubMed PMID: 31555664; PubMed Central PMCID: PMCPMC6742908.
25. Sarbeng EB, Liu Q, Tian X, Yang J, Li H, Wong JL, et al. A functional DnaK dimer is essential for the efficient interaction with Hsp40 heat shock protein. J Biol Chem. 2015;290(14):8849-62. Epub 20150128. doi: 10.1074/jbc.M114.596288. PubMed PMID: 25635056; PubMed Central PMCID: PMCPMC4423677.

26. Trcka F, Durech M, Vankova P, Chmelik J, Martinkova V, Hausner J, et al. Human Stress-inducible Hsp70 Has a High Propensity to Form ATP-dependent Antiparallel Dimers That Are Differentially Regulated by Cochaperone Binding. Molecular & cellular proteomics : MCP. 2019;18(2):320-37. Epub 20181120. doi: 10.1074/mcp.RA118.001044. PubMed PMID: 30459217; PubMed Central PMCID: PMCPMC6356074.
27. Casey AK, Moehlman AT, Zhang J, Servage KA, Kramer H, Orth K. Fic-mediated deAMPylation is not dependent on homodimerization and rescues toxic AMPylation in flies. J Biol Chem. 2017;292(51):21193-204. Epub 20171031. doi: 10.1074/jbc.M117.799296. PubMed PMID: 29089387; PubMed Central PMCID: PMCPMC5743091.
28. Toledo H, Carlino A, Vidal V, Redfield B, Nettleton MY, Kochan JP, et al. Dissociation of glucose-regulated protein Grp78 and Grp78-IgE Fc complexes by ATP. Proc Natl Acad Sci U S A. 1993;90(6):2505-8. doi: 10.1073/pnas.90.6.2505. PubMed PMID: 8460165; PubMed Central PMCID: PMCPMC46116.
29. Carlino A, Toledo H, Skaleris D, DeLisio R, Weissbach H, Brot N. Interactions of liver Grp78 and Escherichia coli recombinant Grp78 with ATP: multiple species and disaggregation. Proc Natl Acad Sci U S A. 1992;89(6):2081-5. doi: 10.1073/pnas.89.6.2081. PubMed PMID: 1532251; PubMed Central PMCID: PMCPMC48600.
30. Hornung P, Maier M, Alushin GM, Lander GC, Nogales E, Westermann S. Molecular architecture and connectivity of the budding yeast Mtw1 kinetochore complex. J Mol Biol. 2011;405(2):548-59. Epub 20101112. doi: 10.1016/j.jmb.2010.11.012. PubMed PMID: 21075115; PubMed Central PMCID: PMCPMC3017645.
31. Westermann S, Drubin DG, Barnes G. Structures and functions of yeast kinetochore complexes. Annual review of biochemistry. 2007;76:563-91. doi: 10.1146/annurev.biochem.76.052705.160607. PubMed PMID: 17362199.
